# Neuropathology in Mouse Models of Mucopolysaccharidosis Type I, IIIA and IIIB

**DOI:** 10.1371/journal.pone.0035787

**Published:** 2012-04-27

**Authors:** Fiona L. Wilkinson, Rebecca J. Holley, Kia J. Langford-Smith, Soumya Badrinath, Aiyin Liao, Alex Langford-Smith, Jonathan D. Cooper, Simon A. Jones, J. Ed Wraith, Rob F. Wynn, Catherine L. R. Merry, Brian W. Bigger

**Affiliations:** 1 Stem Cell & Neurotherapies, Faculty of Medical and Human Sciences, University of Manchester, Manchester, United Kingdom; 2 Stem Cell Glycobiology, School of Materials, University of Manchester, Manchester, United Kingdom; 3 Pediatric Storage Disorders Laboratory, Department of Neuroscience, and Centre for the Cellular Basis of Behaviour, MRC Centre for Neurodegeneration Research, Institute of Psychiatry, King's College London, United Kingdom; 4 Genetic Medicine, St Mary's Hospital, Manchester, United Kingdom; 5 Blood and Marrow Transplant Unit, Royal Manchester Children's Hospital, Manchester, United Kingdom; French National Centre for Scientific Research, France

## Abstract

Mucopolysaccharide diseases (MPS) are caused by deficiency of glycosaminoglycan (GAG) degrading enzymes, leading to GAG accumulation. Neurodegenerative MPS diseases exhibit cognitive decline, behavioural problems and shortened lifespan. We have characterised neuropathological changes in mouse models of MPSI, IIIA and IIIB to provide a better understanding of these events.

Wild-type (WT), MPSI, IIIA and IIIB mouse brains were analysed at 4 and 9 months of age. Quantitative immunohistochemistry showed significantly increased lysosomal compartment, GM2 ganglioside storage, neuroinflammation, decreased and mislocalised synaptic vesicle associated membrane protein, (VAMP2), and decreased post-synaptic protein, Homer-1, in layers II/III-VI of the primary motor, somatosensory and parietal cortex. Total heparan sulphate (HS), was significantly elevated, and abnormally *N*-, 6-*O* and 2-*O* sulphated compared to WT, potentially altering HS-dependent cellular functions. Neuroinflammation was confirmed by significantly increased MCP-1, MIP-1α, IL-1α, using cytometric bead arrays. An overall genotype effect was seen in all parameters tested except for synaptophysin staining, neuronal cell number and cortical thickness which were not significantly different from WT. MPSIIIA and IIIB showed significantly more pronounced pathology than MPSI in lysosomal storage, astrocytosis, microgliosis and the percentage of *2-O* sulphation of HS. We also observed significant time progression of all genotypes from 4–9 months in lysosomal storage, astrocytosis, microgliosis and synaptic disorganisation but not GM2 gangliosidosis. Individual genotype*time differences were disparate, with significant progression from 4 to 9 months only seen for MPSIIIB with lysosomal storage, MPSI with astrocytocis and MPSIIIA with microgliosis as well as neuronal loss. Transmission electron microscopy of MPS brains revealed dystrophic axons, axonal storage, and extensive lipid and lysosomal storage. These data lend novel insight to MPS neuropathology, suggesting that MPSIIIA and IIIB have more pronounced neuropathology than MPSI, yet all are still progressive, at least in some aspects of neuropathology, from 4–9 months.

## Introduction

The series of pathogenic events that cause severe neurodegeneration and ultimately death in the mucopolysaccharide (MPS) diseases is still not fully understood. Amongst this complex set of diseases, MPSI (Hurler), IIIA and IIIB (Sanfilippo) are inherited neurodegenerative lysosomal storage disorders (LSD) caused by deficiency of the glycosaminoglycan (GAG) degrading enzymes α-iduronidase (IDUA), *N*-sulphoglucosamine sulphohydrolase (SGSH) or α-*N*-acetylglucosaminidase (NAGLU) respectively. This deficiency leads to storage of GAGs in all cells of the body causing multisystem disease, with specific phenotypes exhibited depending upon the type of GAG stored. In addition to peripheral disease such as organomegaly, cardiac and respiratory insufficiency, there are MPS sub-types that store dermatan sulphate and chondroitin sulphate and exhibit bone and joint disease (eg MPSI, II, IV, VI and VII), whereas those that store heparan sulphate (HS; eg MPSI, II, III and VII) show severe progressive neurodegeneration [1], [2]. MPSI Scheie, an attenuated non-neuropathic form of MPSI, can be treated using enzyme replacement therapy (ERT). However, patients with a more severe neuropathic form, (MPSI Hurler), require a haematopoietic stem cell transplant (HSCT) where donor-derived cells are able to traffic across the blood-brain barrier and cross-correct cells in the brain. MPSIII patients present with severe behavioural changes such as aggression, hyperactivity and disrupted sleep [3], [4] but HSCT does not ameliorate the brain disease and ERT is unlikely to cross the blood-brain barrier [5], [6]. Substrate reduction therapy (SRT), using high doses of the isoflavone genistein aglycone, has shown very promising results in a mouse model of MPSIIIB [7], [8].

Accumulation of excess HS has the potential to influence many downstream events since this GAG plays a major role in a number of critical processes in the body. HS is essential during development and adult life, where it has a role in the regulation of various crucial signalling pathways by interacting with molecules such as growth factors and morphogens, as well as being an important component of the extracellular matrix [9], [10], [11], [12]. HS structure is often changed during disease, whereby its altered binding properties act to exacerbate the disease phenotype [13], [14]. HS is composed of alternate repeating units of glucosamine (GlcN) and uronic acid (glucuronic acid [GlcA] or iduronic acid [IdoA]), which can be variably sulphated at the *N*-, 6-*O*- and rarely 3-*O*-position of glucosamine and/or 2-*O-*position of uronic acid (NS, 6S, 3S and 2S). These sulphate groups are added in small clusters (sulphated domains), separated by regions with no or little modification (N-acetylated- [NAc−]) domains, with the positioning of sulphate groups ultimately dictating biological function. For example, 6-*O*- and 2-*O*-sulphate groups are essential for the formation and signalling of active fibroblast growth factor/fibroblast growth factor receptor complexes [15], and 2-*O* sulphated HS is also important in HS:SDF-1 (stromal cell derived factor-1) interaction and signalling [16]. Recently we have shown elevated levels of *N*-, 2-*O*- and 6-*O*- sulphated HS in MPSI mouse brain using reverse-phase HPLC separation of AMAC-derivatised disaccharides [17]. Increased *N*-sulphated disaccharides have been detected in MPSIIIA mouse brain [18] and 2-*O*-sulphated HS in MPSI and IIIA patient urine and serum [19], [20] using tandem mass spectrometry.

Mouse models of MPSI and III can be used to test various treatment strategies and to facilitate the understanding of the neurological pathology involved at various stages. It has been shown that MPSI, IIIA and IIIB mouse brains exhibit primary storage of HS, secondary accumulation of GM2 and 3 gangliosides and severe neuroinflammation [6], [21], [22], [23], [24], [25], but these parameters have never been fully quantified so that direct comparisons could be made between these three sub-types in detail. Changes in the level of synaptic proteins, vesicle associated membrane protein 2 (VAMP2 or synaptobrevin 2) and synaptophysin, have been observed in MPSIIIB mice, which may result in altered downstream synaptic signalling events [8], [26], [27], but this has not been shown for MPSI and MPSIIIA. Alterations in the levels of synaptic proteins may result in behavioural changes and ultimately neurodegeneration.

The aim of this study was to examine, compare and fully quantify various neuropathological phenotypes in the brains of MPSI, IIIA and IIIB mice to establish the events that lead to neurodegeneration and provide additional outcome measures for MPS treatment strategies. Brains from mice aged between 4 and 9 months were analysed since we have noted behavioural changes over this time frame [27], [28]. This was achieved by quantitative immunohistochemical analysis for lysosomal compartment size, secondary storage of GM2 ganglioside, neuroinflammation, the synaptic proteins VAMP2, synaptophysin and Homer-1, measurements of cerebral cortical thickness, neuronal cell number and transmission electron microscopy (TEM). Biochemical analysis and quantification of HS amounts and HS sulphation patterning in MPS brain were also analysed, which have not been shown previously for MPSIIIA and IIIB mouse brains. We found that MPSI, IIIA and IIIB mouse brains had extensive primary and secondary storage, as well as significant neuroinflammation, with a loss of organisation of synaptic vesicle membrane protein VAMP2, a reduction in the post-synaptic protein Homer-1 but no change in synaptophysin levels and no decrease in cerebral cortical thickness or overall change in neuronal cell number. Elevated levels of HS were present in MPS brains compared to WT. More importantly, this HS was found to be abnormal, being heavily sulphated along its length, which is likely to alter its biological function. This may provide an insight into understanding the neuropathogenic events that drive severe neurodegeneration and ultimately death.

## Results

### Lysosomal compartment size becomes progressively larger in brains from MPSI, MPSIIIA to MPSIIIB mice

**Figure 1:**
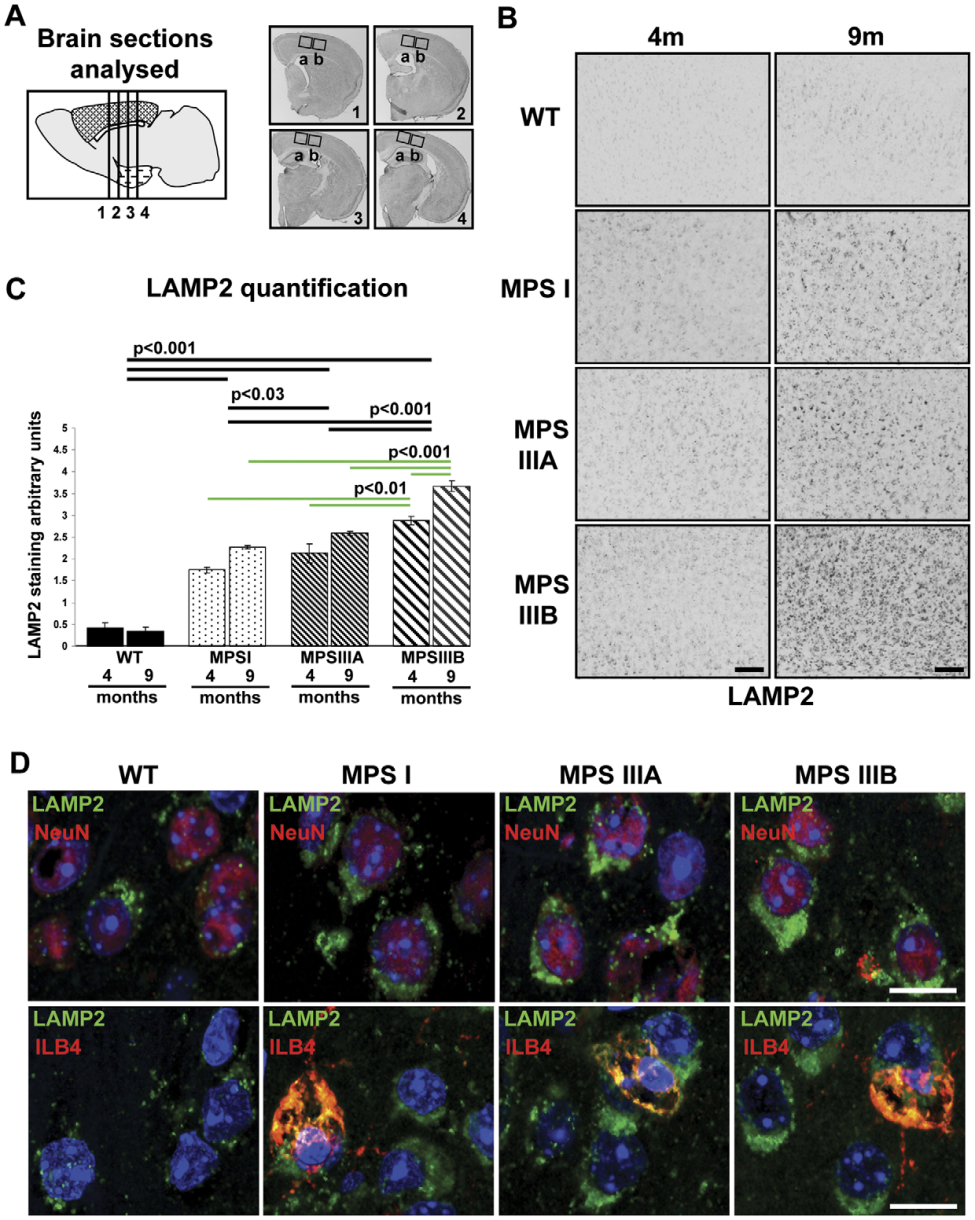
Lysosomal compartment size is significantly increased in MPS brain and localises to neurons and microglia. Lysosomal compartment size was measured by quantifying LAMP2 immunohistochemical staining of WT, MPSI, IIIA and IIIB mouse cerebral cortex at 4 and 9 months of age (4 m and 9 m; n = 3 mice per group). (A) Four sections of brain from Bregma 0.26, −0.46 −1.18 and −1.94 mm were stained concurrently. (B) Representative sections stained with LAMP2 that correspond to a whole field of view covering cortical layers II/III–VI (Section 2a in A). Bars = 100 µm. (C) Two fields of view (boxed areas in A; ×20 objective) from each section were quantified using ImageJ. Error bars represent the SEM and p values are from two way ANOVA with Tukey's multiple comparisons test. Significant overall genotype differences are denoted by thick black lines. Individual genotype*time differences are shown by thin green lines. The significant individual genotype*time differences (p<0.001) between all MPSs and WT at each time point are not shown for clarity. (D) LAMP2 (green) was detected in NeuN-positive neurons (red) and in ILB4-positive microglia (red) in layer II/III of WT, MPSI, IIIA and IIIB cerebral cortex. Nuclei are stained with DAPI (blue); Bar = 10 µm. Single colours and overlays are shown in Figure S1.

Quantification of LAMP2 (lysosomal associated membrane protein) immunoreactivity from 2 fields of view each from 4 sections per mouse (Figure 1A) was used to compare the size of the lysosomal compartment in the primary motor, somatosensory and parietal areas of the cerebral cortex in WT, MPSI, IIIA and IIIB at 4 and 9 months of age. LAMP2 immunoreactivity exhibited an intense vesicular staining pattern that was more intense in MPS brain than WT (Figure 1B, C and D). Two way ANOVA for genotype versus time revealed a significant genotype effect, with LAMP2 staining in MPSIIIB significantly increased over MPSIIIA (p<0.001), MPSI (p<0.001) and WT (p<0.001), MPSIIIA significantly increased over both MPSI (p<0.03) and WT (p<0.001) and MPSI significantly increased over WT (p<0.001; Figure 1C). There was also a significant overall time effect, with 9 months LAMP2 staining more intense than 4 months (p<0.001). However, the genotype*time interaction was also significant (p<0.01) suggesting that different genotypes change differentially over time. Where significant genotype*time effects were seen, we established that WT was the genotype behaving differently to the MPS genotypes by performing a confirmatory 2 way ANOVA on time vs genotype for MPS genotypes alone. This allowed us to confirm that MPS genotypes all progress over time for LAMP2. When multiple comparisons were made between all genotypes at all times, (green lines), each WT group had significantly less LAMP2 staining than any MPS group (p<0.001; not shown on figure). In MPSIIIB brains, the lysosomal compartment became progressively larger from 4 months to 9 months (p<0.001; Figure 1C) and was also significantly larger than both MPSI and IIIA at 4 (p<0.01) and 9 months of age (p<0.001; Figure 1C).

In all other regions of MPS brain, LAMP2 staining was much more intense than in WT and was observed in various cell types in MPSI, IIIA and IIIB. Confocal microscopy at a higher magnification confirmed our observations and showed an enlarged lysosomal compartment (LAMP2) in both neurons (neuronal nuclei; NeuN) and in microglia (Isolectin B4; ILB4) in layer II/III as shown in representative sections of MPSI, IIIA and IIIB cerebral cortex (Figure 1D; Single colours and overlays are shown in Figure S1).

**Figure 2:**
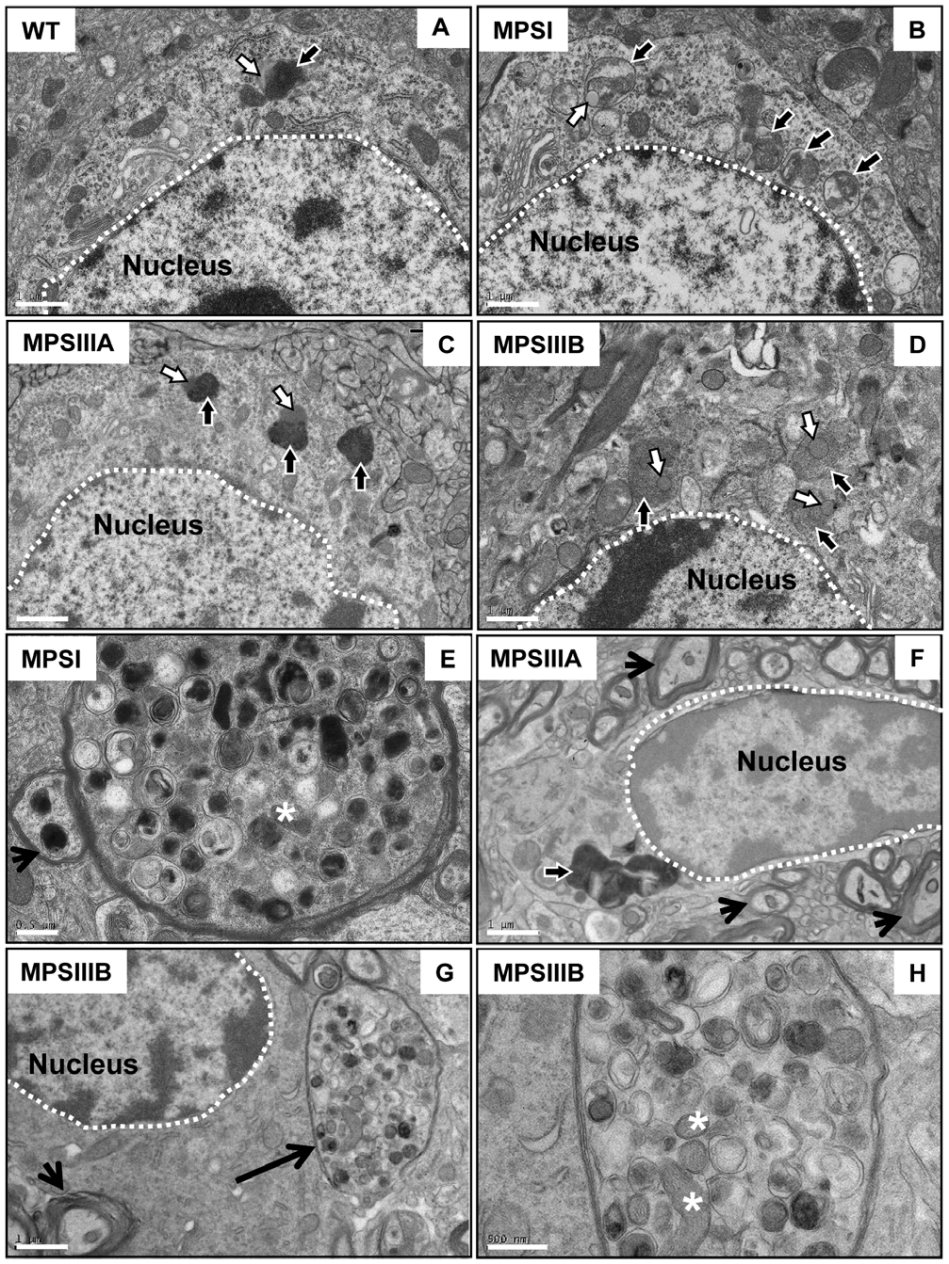
Transmission electron microscopic analysis of MPS brain showing an increase in lysosomal burden in MPS cerebral cortex and large dystrophic axons in MPSI and IIIB. Images show an increase in lysosomal burden (black arrows outlined in white) in MPSI, IIIA and IIIB (B–D and F; Bars = 1 µm) compared to WT cerebral cortex (A; Bar = 1 µm). Lipid (white arrows outlined in black) is also stored in the lysosomes of MPS brain (B, C, and D) and also a small amount in WT (A). Dystrophic axons were observed in MPSI (E; Bar = 0.5 µm) and IIIB (arrow in G; Bar = 1 µm and enlarged in H; Bar = 0.5 µm) cerebral cortex but not in MPS IIIA (F; Bar = 1 µm). These structures contained organelles similar to immature and mature autophagosomes with electron dense material and some mitochondria (*; E and H). Normal axons were also observed in all MPS types (E, F and G; arrow heads).

Transmission electron microscopy (TEM) revealed an increase in lysosomal storage burden in the cells of the cerebral cortex in MPS brain compared to WT (black arrows outlined in white; Figure 2A–D, F). Most of the lysosomes also exhibited lipid storage as shown in Figure 2A–D (white arrows outlined in black). MPS brain cells also contained many more vacuoles and the cytoplasm appeared to be denser than that observed in WTs. In addition, dystrophic axons that contained storage material were observed in MPSIIIB cerebral cortex (Figure 2G and H) at a much greater frequency than in MPSI (Figure 2E), but were not detected in any of the MPSIIIA brain sections examined (n = 3 per group; Figure 2F). These dystrophic axons contained multiple vesicular organelles, some of which exhibited very electron dense material, were similar to immature and mature autophagosomes, and some also contained mitochondria (denoted by* in Figure 2E and H). Notably, the thickness of myelination in these dystrophic axons was substantially reduced compared to WT. All MPS types also exhibited normal axons (Figure 2E–G; arrow heads).

### MPSI, IIIA and IIIB brains exhibited a significant increase in the amount and level of sulphation of HS disaccharides compared to WT

To investigate the accumulation and structure of stored HS GAGs, HS chains were purified from WT, MPSI, IIIA and IIIB mouse brain tissue (1 hemisphere per mouse at 9 months of age) and completely depolymerised into their component disaccharides using bacterial heparinase enzymes. Reverse-phase HPLC separation of AMAC-derivatised disaccharides was then used to quantify the amounts and type of sulphation patterns in each HS type, using integration analysis of peak area to enable quantification.

**Figure 3:**
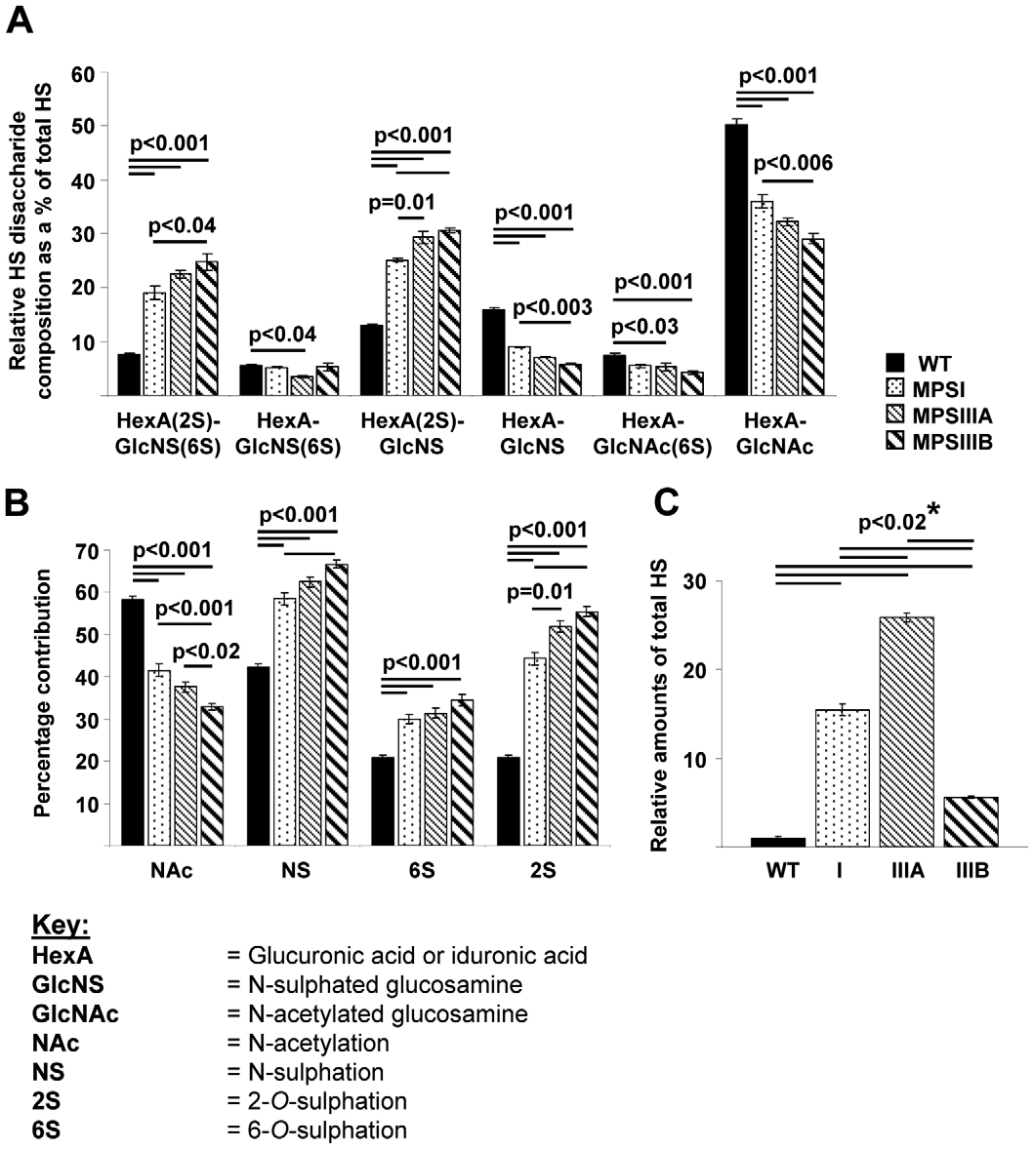
Significant increases in the amount and sulphation of HS in MPS brains. (A) Compositional disaccharide analysis of HS from WT, MPSI, MPSIIIA and MPSIIIB brains. (B) *N*-acetlylation, *N*-, 6-*O*-, and 2-*O*-sulphate distribution within HS isolated from WT, MPSI, MPSIIIA and MPSIIIB brains. Values are calculated from the disaccharide analyses shown in A, specifying the percentage of total disaccharides along the HS chain that contain each modification. (C) Total relative amounts of HS. * denotes where all groups were significantly different from each other. n = 3 mice per group, error bars represent the SEM and p values are from one way ANOVA with Tukey's multiple comparisons test.

HS composition analysis revealed that all three MPS types, exhibited significantly increased levels of tri-sulphated disaccharide HexA(2S)-GlcNS(6S) (where HexA is GlcA or IdoA), typically confined to the most highly sulphated domains within HS, compared to WT (p<0.001) by 2.5, 2.9 and 3.2 fold respectively (Figure 3A). There was also a significant increase in these tri-sulphated disaccharides in MPSIIIB compared to MPSI (p<0.04), but not between MPSI and IIIA or IIIA and IIIB (Figure 3A). A similar result was also observed for HexA(2S)-GlcNS (Figure 3A) with MPS brain exhibiting significantly increased levels compared to WT (p<0.001) by around 2 to 2.4 fold. A significant increase in HexA(2S)-GlcNS in MPSIIIB compared to MPSI (p<0.001) and MPSIIIA compared to MPSI (p = 0.01) was also visible, but was not seen between MPSIIIA and IIIB.

As expected, the opposite effect was observed with mono-sulphated or non-sulphated HS disaccharides, with a significant decrease in the levels of HexA-GlcNS and HexA-GlcNAc disaccharides in MPSI, IIIA and IIIB compared to WT (p<0.001; Figure 3A), with a significant reduction in HexA-GlcNS (p<0.003) and HexA-GlcNAc (p<0.006) from MPSI to IIIB, but not between MPSI and IIIA or between IIIA and IIIB. A significant reduction in the level of HexA-GlcNS(6S) was only observed between WT and MPSIIIA (p<0.04) and in HexA-GlcNAc(6S) between WT and MPSIIIA (p<0.03) and IIIB (p<0.001).

Comparing the overall levels of particular HS modifications (by calculating the percentage of total disaccharides containing either GlcNAc or GlcNS, or 2S or 6S groups) highlighted the significant increase in NS, 6S and 2S modification of HS in MPSI, IIIA and IIIB brain (p<0.001) compared to WT, with a general trend of increasing levels of all types of sulphation modification from MPS I through to IIIA then IIIB (Figure 3B). This was reflected by a significant reduction in unmodified NAc containing disaccharides compared to WT (p<0.001; Figure 3B). No significant differences were observed between MPSI and IIIA, but a significant reduction was found between MPSI and IIIB (p<0.001) and MPSIIIA and IIIB (p<0.02). The most significantly increased modification was in 2S. In WT brain only 20% of the total disaccharides in the HS chain were modified by the addition of a 2S group. However this increased to 44% in MPSI, 52% in IIIA and 56% in IIIB. Significant differences were observed between MPSI and IIIA (p = 0.01) and MPSI and IIIB (p<0.001). 2S is typically restricted to sulphated domains, implying large increases in either the number or length of sulphated domains within the HS chain.

This method of analysis also enabled the relative levels of total HS to be compared quantitatively between WT and MPS types [29]. Compared to WT, a significant 15.5-fold increase in the amount of HS was apparent in MPSI mice, increasing to ∼26-fold in MPSIIIA (Figure 3C). Interestingly, although levels of HS were markedly increased in MPSIIIB compared to WT by ∼5.5-fold (p<0.02), this increase was significantly less than amounts seen in MPSI and MPSIIIA (p<0.02). This suggests that despite the fact the HS in MPSIIIB mice is abnormally highly sulphated, less is stored in the brain than the other MPS types.

### A significant amount of secondary storage of gangliosides occurs by 4 months of age in MPSI, IIIA and IIIB mouse brains

**Figure 4:**
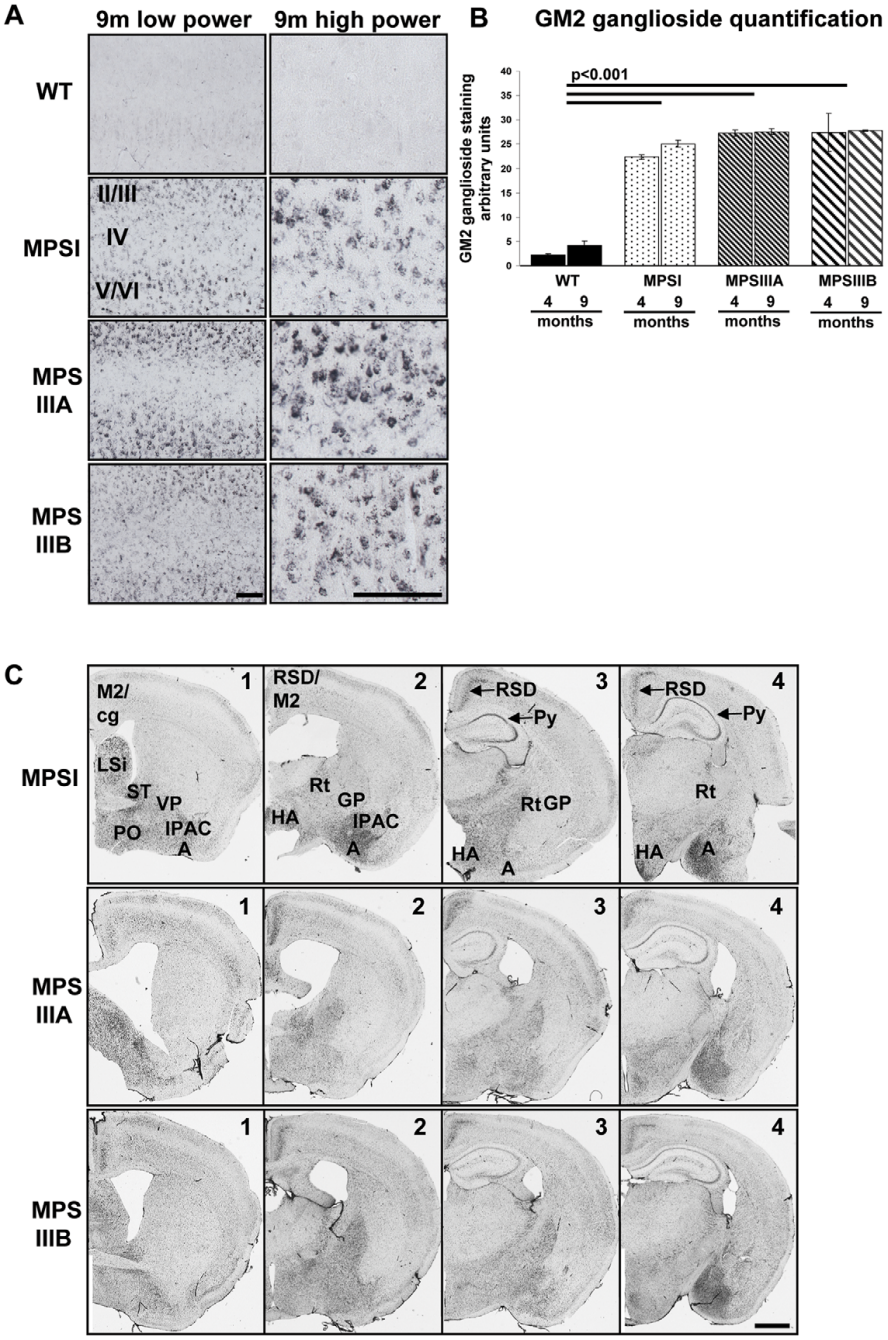
Secondary storage of GM2 gangliosides in MPS mouse brain. (A) Representative images of low (cerebral cortical layers II/III–VI) and high power sections (cerebral cortical layers II/III) of GM2 ganglioside stained sections at 9 months of age (9 m). Bars = 100 µm. (B) Four sections of brain (Bregma 0.14, −0.58, −1.34, −2.2 mm) were stained concurrently for GM2 gangliosides and images of two low power (×20 objective) fields of view covering cortical layers II/III–VI (boxed areas, Figure 1A; whole field of view of section 2a is shown in Figure 4A low power) were captured from each section and quantified using ImageJ (n = 3 mice per group). Error bars represent the SEM and p values are from two way ANOVA with Tukey's multiple comparisons test. Significant overall genotype differences are denoted by thick black lines. (C) Low power plans (×2.5 objective) of representative sections of a brain analysed from each group, showing the location of the very intense GM2 immunoreactivity. These areas are labelled as secondary motor cortex (M2), amygdala (A), cingulum (cg), lateral septal nucleus (LSi), stria terminalis (ST), preoptic area (PO), ventral pallidum (VP), globus pallidus (GP), interstitial nucleus of the posterior limb of the anterior commisure (IPAC), retrosplenial granular cortex (RSD), thalamic (Rt) and hypothalamic (HA) areas and pyramidal cells of the hippocampus (Py). Bar = 100 µm.

To reveal the secondary storage of accumulated GM2 gangliosides in MPS brain, GM2 ganglioside immunoreactivity was performed and quantified across layer II to V/VI of the primary motor, somatosensory and parietal areas of the cerebral cortex, as shown in the low power images in Figure 1A (8 fields of view per mouse; n = 3). GM2 ganglioside immunoreactivity in MPS mice exhibited an intense vesicular staining pattern in layers II/III and V/VI of the cerebral cortex with less staining in layer IV and was negligible in WT (Figure 4A [low power of layers II/III to VI and high power of layer II/III]). Two way ANOVA for genotype versus time revealed a significant genotype effect with GM2 ganglioside staining in MPS brain significantly increased over WT (p<0.001). There were no significant differences between the MPS genotypes and no overall time effect (Figure 4A and B).

Intense GM2 staining was also found in other areas of MPS brain in addition to the cerebral cortex, with extremely intense GM2 immunoreactivity readily observable at low magnification (Figure 4C) and was consistent between the three MPS types. As stated above, negligible staining was seen in the WT brain (data not shown). Very intense GM2-immunoreactivity was observed in the following structures; secondary motor cortex (M2), amygdala (A), lateral septal nucleus (LSi), stria terminalis (ST), preoptic area (PO), ventral pallidum (VP), interstitial nucleus of the posterior limb of the anterior commisure (IPAC), retrosplenial granular cortex (RSD), hypothalamic (HA) areas and the pyramidal cells of the hippocampus (Pyr); with relatively less intense staining in the cingulum (cg), thalamic area (Rt) and globus pallidus (GP), (Figure 4C).

### MPSI, IIIA and IIIB mouse models exhibit chronic neuroinflammation

**Figure 5:**
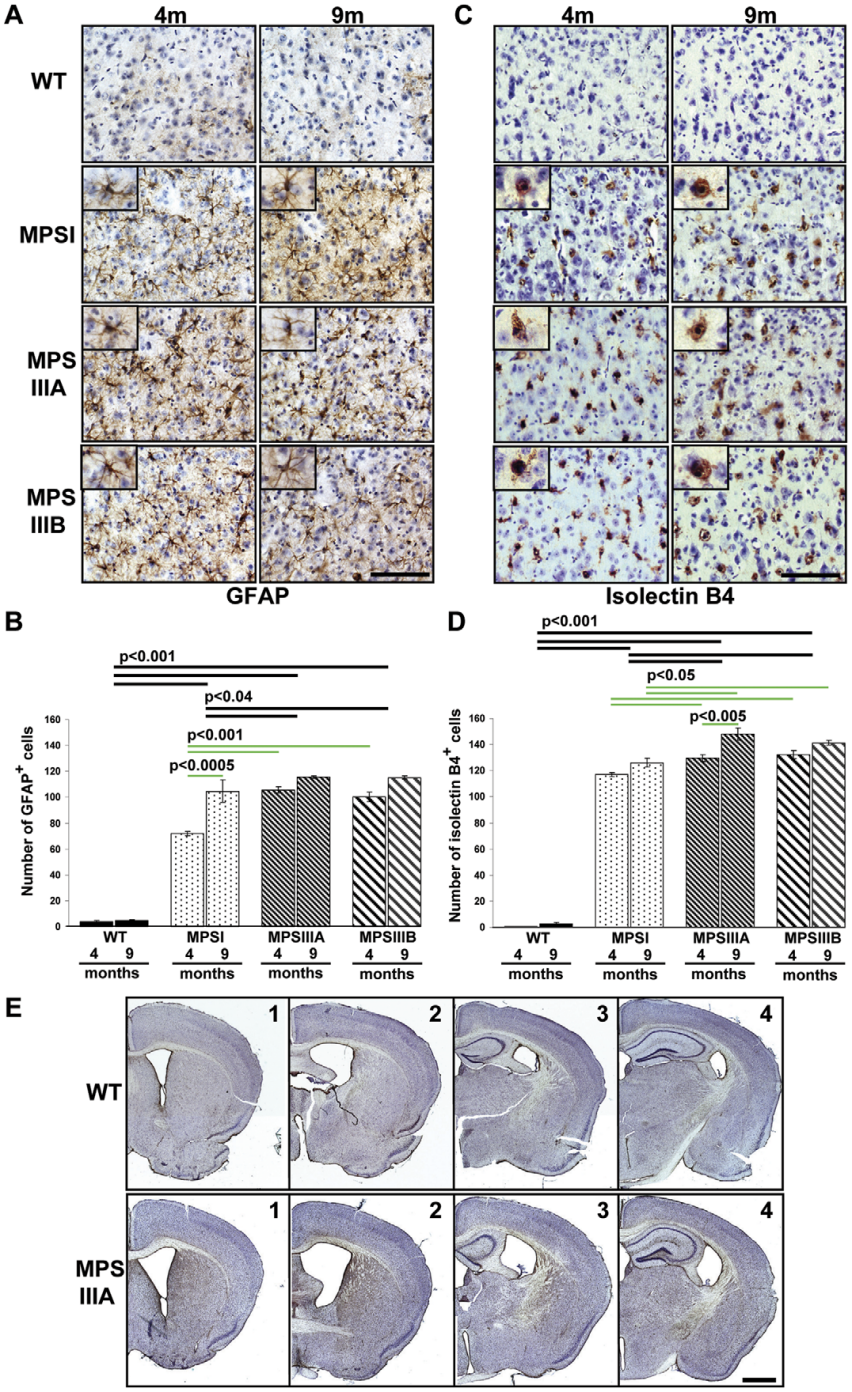
Significant neuroinflammation in MPS cerebral cortex at 4 and 9 months of age. Representative sections of positively stained (A) astrocytes (GFAP; brown) and (C) microglia (Isolectin B4:brown) at 4 and 9 months of age (4 m and 9 m) that correspond to a high power view covering cortical layer IV (from section 2a, Figure 1A). Boxed inserts show an enlarged single positively stained astrocyte or microglial cell. Sections were counterstained with Mayer's haematoxylin to highlight the nuclei. Bar = 100 µm. Whole fields of view used for counting are shown in Figures S2 and S3. Four sections of brain were stained concurrently with either GFAP or Isolectin B4, and images of two low power fields of view covering cortical layers II/III–VI (boxed areas, Figure 1A) were captured from each section. The number of GFAP or Isolectin B4 positive cells in these areas were counted using ImageJ and expressed as an average number of cells per section (B and D respectively; n = 3 mice per group). Error bars represent the SEM and p values are from two way ANOVA with Tukey's multiple comparisons test. Significant overall genotype differences are denoted by thick black lines. Individual genotype*time differences are shown by thin green lines. The significant individual genotype*time differences (p<0.001) between all MPSs and WT at each time point are not shown for clarity. (E) Low power plans (×2.5 objective) showing 4 representative brain sections from WT and MPS IIIA mice stained with Isolectin B4. Sections from MPSI and MPSIIIB are not shown but are virtually identical in distribution and intensity. Bar = 100 µm.

Astrocyte activation was assessed by counting the number of GFAP-positive astrocytes from 8 fields of view per mouse (n = 3) from the primary motor, somatosensory and parietal areas of the cerebral cortex as shown in Figure 1A. Two way ANOVA for genotype versus time revealed a significant overall genotype effect with astrocyte activation in MPSIIIB and IIIA significantly increased over MPSI (p<0.04) and WT (p<0.001), and all MPS genotypes were significantly increased over WT (p<0.001; Figure 5A and B). There was also a significant overall time effect, with 9 months astrocyte activation higher than 4 months (p<0.001). However, the genotype*time interaction was also significant (p<0.01) suggesting that different genotypes change differentially over time. Where significant genotype*time effects were seen, we established that WT was the genotype behaving differently to the MPS genotypes by performing a confirmatory 2 way ANOVA on time vs genotype for MPS genotypes alone. This allowed us to confirm that MPS genotypes all progress over time for GFAP.

When multiple comparisons were made between all genotypes at all times (green lines), each WT group had significantly fewer reactive astrocytes than any MPS group (p<0.001; not shown on figure). Astrocyte activation in MPSI increased from 4 months to 9 months of age (p<0.0005; Figure 5B), and was lower at 4 months compared to MPSIIIA and IIIB (p<0.001). However no significant differences were seen in the level of astrocyte activation between any MPS types at 9 months. In other regions of WT brain, fibrous astrocytes were found in and along the corpus callosum, optic chiasm and along the third ventricle and hippocampus, but only very few palely stained protoplasmic astrocytes were found scattered throughout the cerebral cortex (data not shown). However, in MPS brains, astrocytes were much greater in number throughout each whole section of the brain examined.

Similarly, isolectin B4-positive cells were counted in the same areas to determine the level of microglial activation (Figure 5C and D) in the primary motor, somatosensory and parietal areas of the cerebral cortex as shown in Figure 1A. Two way ANOVA for genotype versus time revealed a significant genotype effect with microglial activation in MPSIIIB and IIIA, significantly increased over both MPSI (p<0.001) and WT (p<0.001), and MPSI was significantly increased over WT (p<0.001; Figure 5C and D). There was also a significant overall time effect, with 9 months microglial activation higher than 4 months (p<0.001).

When multiple comparisons were made between all genotypes at all times (green lines), each WT group had significantly fewer microglia than any MPS group (p<0.001). MPSI brain exhibited significantly less microglial activation than both MPSIIIA and IIIB at each time point (p<0.05) with no significant differences between MPSIIIA and IIIB at either time point. Microglial activation in MPSIIIA was found to significantly increase over time (p<0.005; Figure 5D).

Very few isolectin B4-stained microglial cells were found in these areas of WT brains, with only 1 or 2 cells detected per field of view in the cerebral cortex and optic chiasm, otherwise the sections contained no positively stained microglia (Figure 5C and E). In contrast, in MPS brains, microglia were much larger and evenly distributed across the entire brain section (sections 1–4; Figure 5E, representative low power images for MPSIIIA) at a density similar to that observed in the cerebral cortex (Figure 5C) with a limited number in the lateral preoptic area. Examples of whole fields of view corresponding to section 2a that were used for counting are included in Figure S2 and S3 for GFAP and ILB4 respectively.

**Figure 6:**
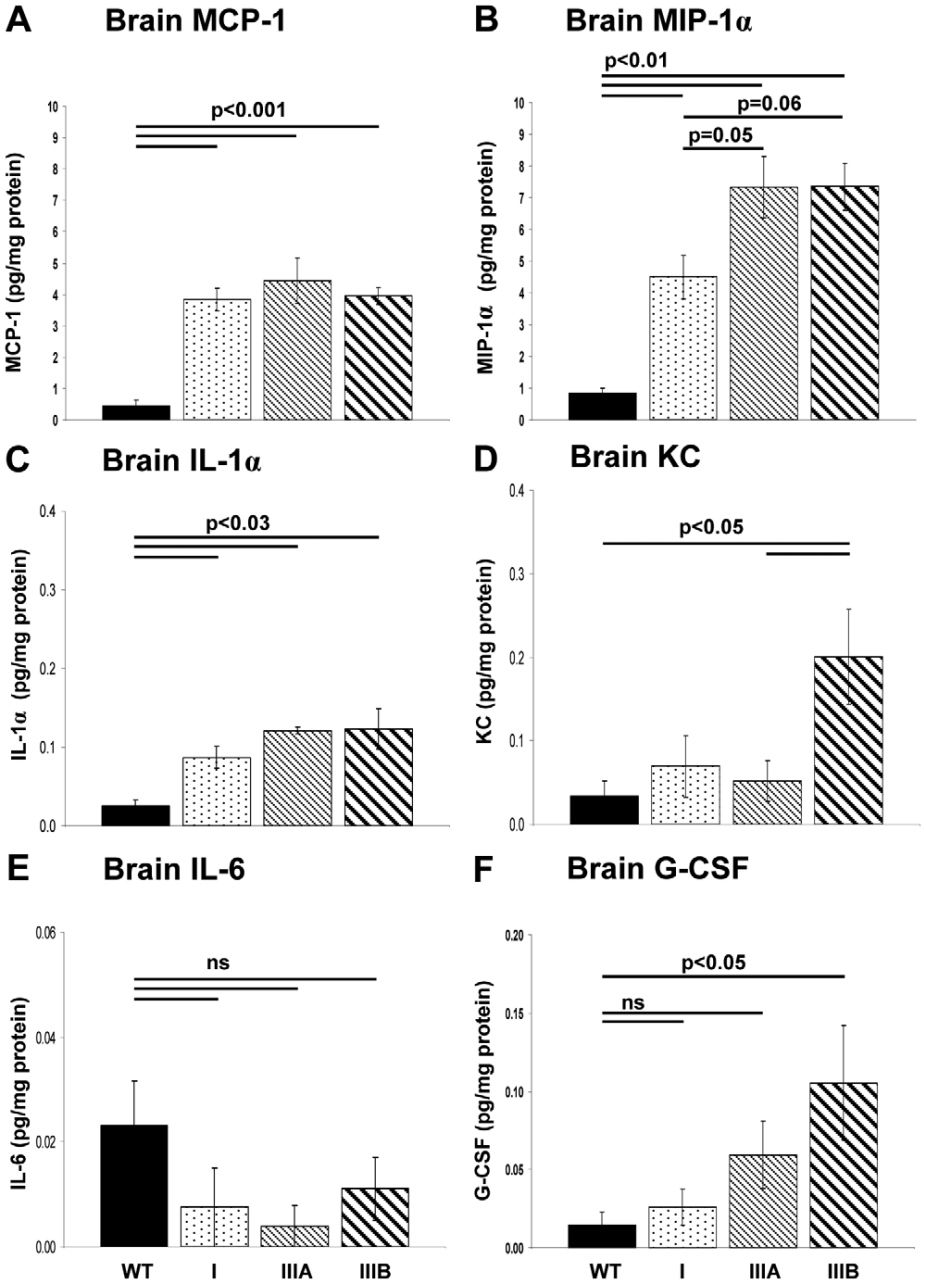
Elevated inflammatory cytokines in MPS at 9 months of age. WT, MPSI, IIIA and IIIB mouse brains were analysed using cytometric bead array analysis to quantify a set of inflammatory cytokines isolated from whole extracts to further quantify the level of neuroinflammation in MPS (A) MCP-1, (B) MIP-1α (C) IL-1α, (D), KC (E) IL-6 and (F) G-CSF. IFNγ, IL-1β, IL-3, IL -9, IL-13 and GM-CSF were below the level of detection using this assay. n = 5–6 mice per group, error bars represent the SEM and p values are from one way ANOVA with Tukey's multiple comparisons test.

Cytometric bead array analysis (CBA) was used to quantify a set of inflammatory cytokines isolated from extracts of whole brain from WT, MPSI, IIIA and IIIB mice at 8–9 months of age (n = 5–6 per group) to further quantify the level of neuroinflammation in MPS. Significant increases of monocyte chemoattractant protein (MCP-1/CCL2; p<0.001), macrophage inflammatory protein (MIP-1α/CCL3; p<0.01) and interleukin-1α (IL-1α; p<0.03) were detected in brains of MPSI, IIIA and IIIB compared to WT (Figure 6A, B and C). Furthermore, MPSIIIA brain exhibited significantly higher levels of MIP-1α compared to MPSI brain (p = 0.05) and MPSIIIB brain exhibited almost significantly higher levels of MIP-1α compared to MPSI brain (p = 0.06; Figure 6B).

MPSIIIB brain showed significantly elevated KC/CXCL1 levels compared to WT and IIIA (p<0.05), but no differences were observed between WT, MPSI and IIIA brains (Figure 6D). No significant differences in the level of IL-6 was found between WT and MPSI, IIIA and IIIB brain, although there was a trend towards less IL-6 in MPSI, IIIA and IIIB compared to WT (Figure 6E). MPSIIIB brain demonstrated significantly higher levels of G-CSF (granulocyte colony stimulatory factor) compared to WT but no significant differences were found between WT and MPSI or IIIA or between MPSI, IIIA and IIIB. However there was a trend towards an increase from WT to MPSI, IIIA with IIIB mice exhibiting the highest amount (Figure 6F). The levels of IFNγ, IL-1β, IL-3, IL -9, IL-13 and GM-CSF (granulocyte macrophage colony stimulatory factor) were below the level of detection in WT, MPSI, IIIA or IIIB brains using this assay (data not shown).

### No changes in cerebral cortical thickness and neuronal loss in MPS mouse brain

**Figure 7:**
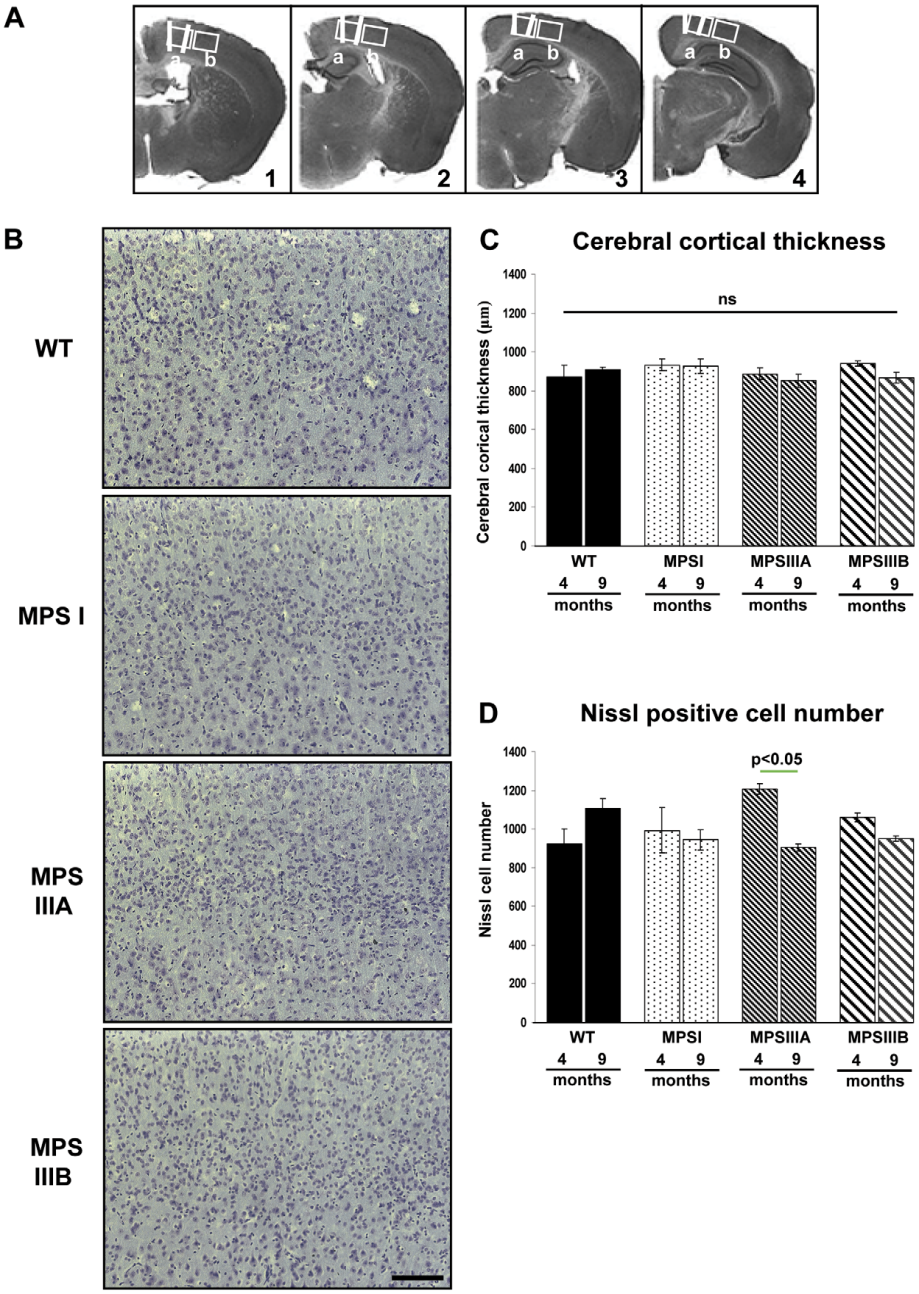
No significant changes in the cortical thickness or Nissl positive cell number. (A) Cerebral cortical thickness was measured from the apex of the cingulum of the corpus callosum to the outside of cerebral cortical layer II. A second measurement was taken 1000 µm laterally from the apex of the cingulum, from the corpus callosum to the outside of cerebral cortical layer II. These measurements (white lines in A) were taken from four sections of brain (Bregma 0.26, −0.46 −1.18 and −1.94 mm) and analysed (C). Four sections of brain (Bregma 0.26, −0.46 −1.18 and −1.94 mm) were stained for Nissl substance (B) and two low power fields of view, covering cortical layers II/III–VI (boxed areas in A), were captured from each section and the number of Nissl positive cells were counted using ImageJ (D). n = 3 mice per group, error bars represent the SEM and p values are from two way ANOVA with Tukey's multiple comparisons test. Significant individual genotype*time differences are shown by a thin green line. Bar = 100 µm.

Two measurements of cortical thickness were taken from each brain section (8 measurements per mouse). The first was from the apex of the cingulum of the corpus callosum to the outside of cerebral cortical layer II and the second was taken 1000 µm laterally from the apex of the cingulum, from the corpus callosum to the outside of cerebral cortical layer II. No significant genotype or time differences were found between the cortical thickness of WT and MPS mice (Figure 7A and C; white lines). Nissl-stained cells were also counted in the primary motor, somatosensory and parietal areas of the cerebral cortex, as shown in Figure 1A and 7A and B. Although no overall significant differences were found in neuronal cell numbers between WTs and MPS types, there was a significant genotype*time effect (p<0.009) with a significant reduction in MPSIIIA from 4 to 9 months (p<0.05) (Figure 7D). Given no significant difference to WT, this result should be treated with caution.

### Alteration of the synaptic proteins, VAMP2 and Homer-1 in MPSI, IIIA and IIIB mouse models

**Figure 8:**
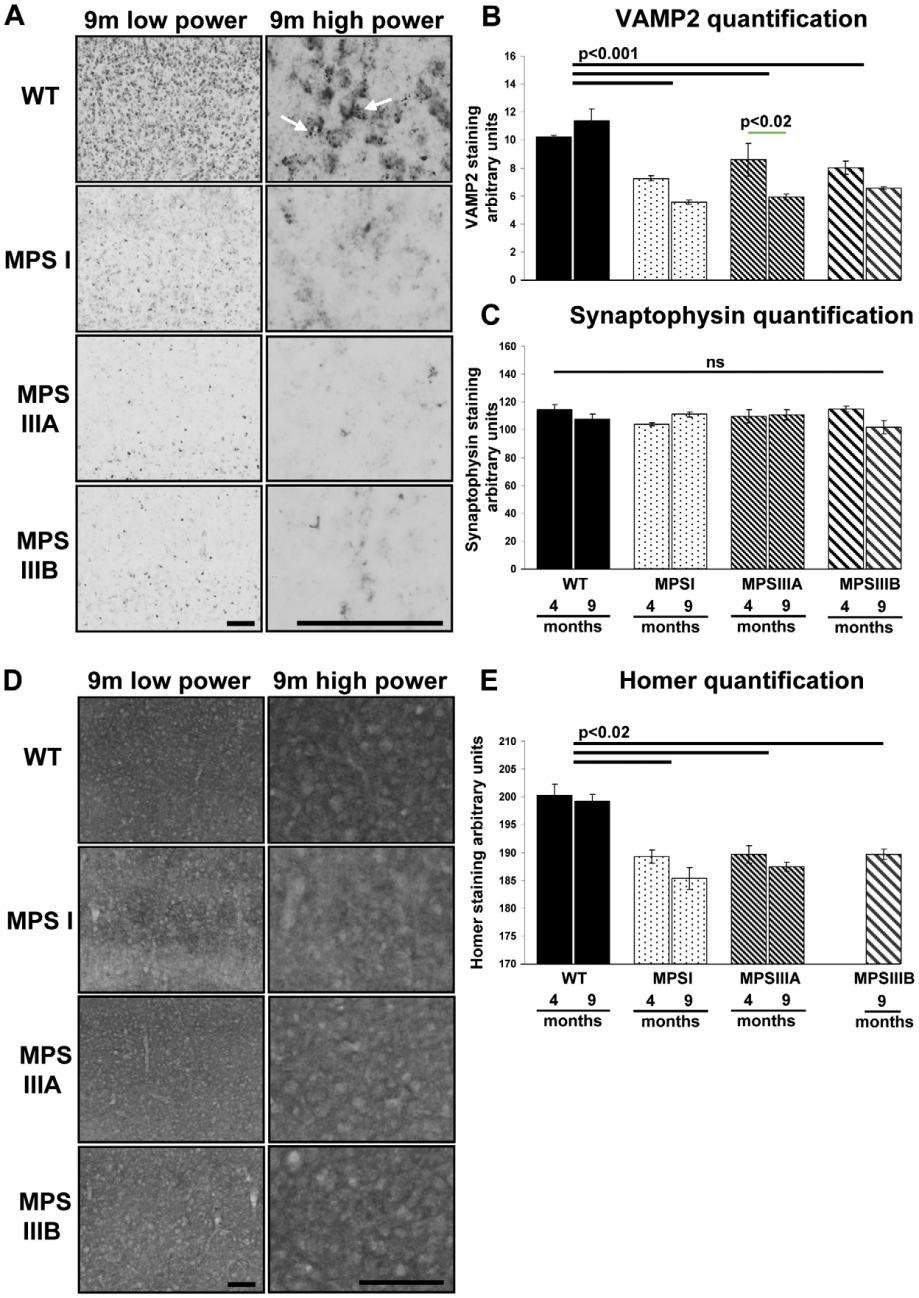
Significantly reduced levels of VAMP2 and Homer-1 and a change in the localisation of VAMP2 in MPS mouse cerebral cortex. Representative images of low (cerebral cortical layers II/III–VI) and high power sections (cerebral cortical layers II/III) of VAMP2 (A) and Homer-1 (D) stained sections at 9 months of age (9 m). WTs exhibit discrete VAMP2 punctate staining (A; white arrows) which is lost in MPSI, IIIA and IIIB mouse brain. Bar = 100 µm. Four sections of brain (Bregma 0.26, −0.46 −1.18 and −1.94 mm) were stained concurrently for VAMP2 (B), synaptophysin (C) and Homer-1 (E) and images of two low power fields of view covering cortical layers II/III–VI (boxed areas, Figure 1A) were captured from each section and quantified using ImageJ (n = 3 mice per group). Error bars represent the SEM and p values are from two way ANOVA with Tukey's multiple comparisons test. Significant overall genotype differences are denoted by thick black lines and individual genotype*time differences are shown by thin green lines. The significant individual genotype*time differences (p<0.001) between all MPSs and WT at each time point are not shown for clarity.

To determine if there was any evidence for synaptic pathology in MPS brains, immunostaining for pre-synaptic vesicle membrane proteins which are involved in the formation of the SNAP/SNARE complex, VAMP2 and synaptopysin, were quantified. VAMP2 exhibited a very distinct vesicular punctate staining in WT cerebral cortex (Figure 8A). However, in MPS cerebral cortex, this punctate staining was lost and was much more diffuse (Figure 8A). VAMP2 staining was quantified in the primary motor, somatosensory and parietal areas of the cerebral cortex (Figure 1A and 8B). Two way ANOVA for genotype versus time revealed a significant genotype effect with VAMP2 staining in MPS brain significantly decreased over WT (p<0.001; Figure 8B). There was a significant overall time effect, with 9 months VAMP2 staining less intense than 4 months (p<0.01). There were no significant differences between the MPS genotypes. The genotype*time interaction was also significant (p = 0.04) suggesting that different genotypes change differentially over time. Where significant genotype*time effects were seen, we established that WT was the genotype behaving differently to the MPS genotypes by performing a confirmatory 2 way ANOVA on time vs genotype for MPS genotypes alone. This allowed us to confirm that MPS genotypes all progress over time for VAMP2. When multiple comparisons were made between all genotypes at all times (green lines), VAMP2 staining was found to be significantly reduced in all MPS groups compared to WT groups (p<0.001; not shown on figure). No significant differences were found in immunoreactivity for VAMP2, between MPSs at either time point, but VAMP2 staining was found to have decreased over time in MPSIIIA (p<0.02); Figure 8B). This relative loss of punctate VAMP2 staining was detected throughout the MPS brain sections examined. Synaptophysin staining was also quantified in the primary motor, somatosensory and parietal areas of the cerebral cortex, but no significant differences were observed between WT and MPS brains at either time point (Figure 8C), suggesting that the altered VAMP2 staining represents a rearrangement of the pre-synaptic compartment rather than an overt loss of synapses.

Homer-1, a protein enriched at the post-synaptic density of excitatory synapses, exhibited a much more diffuse pattern of staining compared to VAMP2 (Figure 8D). Quantification of Homer-1 staining in the primary motor, somatosensory and parietal areas of the cerebral cortex (Figure 1A) showed that it was significantly reduced in MPS brain compared to WTs (p<0.02; Figure 8E). No significant differences were found in immunoreactivity for Homer-1 between MPSs at either time point (data unavailable for MPSIIIB at 4 months). This suggests that signalling at the post-synaptic density may also be altered.

## Discussion

Our data directly compares the severity of neuropathology in terms of primary and secondary storage material, neuroinflammation and the level of two pre-synaptic vesicle proteins and a post-synaptic protein in mouse models of MPSI, IIIA and IIIB. The progression of brain pathology was analysed between 4 and 9 months of age as we have noted behavioural changes over this time frame [27], [28]. The complete set of comparative neuropathology results are summarised in Table 1.

**Table 1:** Summary of MPS comparative neuropathology.

Genotype/Age	WT 4 m	WT 9 m	MPSI 4 m	MPSI 9 m	MPSIIIA 4 m	MPSIIIA 9 m	MPSIIIB 4 m	MPSIIIB 9 m
**LAMP2** *	0.42	0.35	1.75	2.27	2.13	2.59	2.89	3.67
**HS – % NAc**	ND	58.4	ND	41.5	ND	37.5	ND	32.8
**HS – % NS**	ND	42.2	ND	58.5	ND	62.5	ND	66.6
**HS – % 6S**	ND	20.8	ND	29.9	ND	31.4	ND	34.4
**HS – % 2S**	ND	20.7	ND	44.2	ND	51.8	ND	55.4
**Total HS (relative to WT)**	ND	1	ND	15.5	ND	25.8	ND	5.6
**GM2 Ganglioside** *	2.2	4.2	22.3	25.1	27.3	27.5	27.4	27.8
**GFAP (No)**	4	5	72	105	106	116	100	115
**Isolectin B4 (No)**	1	3	117	126	129	148	132	141
**MIP-1α (pg/mg)**	ND	0.8	ND	4.5	ND	7.3	ND	7.4
**MCP-1 (pg/mg)**	ND	0.5	ND	3.8	ND	4.45	ND	4
**IL-1α (pg/mg)**	ND	0.03	ND	0.09	ND	0.12	ND	0.12
**KC (pg/mg)**	ND	0.03	ND	0.07	ND	0.05	ND	0.2
**IL-6 (pg/mg)**	ND	0.023	ND	0.008	ND	0.004	ND	0.011
**G-CSF (pg/mg)**	ND	0.01	ND	0.03	ND	0.06	ND	0.11
**Cortical Thickness (µm)**	871	911	933	929	887	852	940	869
**Nissl (No)**	922	1104	993	945	1207	904	1060	950
**VAMP2** *	10.2	11.4	7.2	5.6	8.6	5.9	8	6.6
**Synaptophysin** *	114	107	104	111	110	111	115	102
**Homer** *	200	199	189	185	190	187	ND	190

* denotes arbitray units; ND denotes not determined; No denotes number.

The lysosomal compartment became progressively larger in MPSI, MPSIIIA and MPSIIIB mouse brains and was significantly different in each case. The difference between MPSIIIA and IIIB may be due in part to the MPSIIIA mouse model retaining around 3–4% of normal SGSH enzyme activity [22], thus it could be a more attenuated model. An enlarged lysosomal compartment has been observed previously in separate studies using mouse models of MPSI [25], MPSIIIA [30] and MPSIIIB [7], [8], [25], [27], but they have not been compared directly with each other in one comprehensive fully quantified study. Interestingly we also observed an overall progression in lysosomal size from 4 to 9 months in all genotypes despite the presence of the WT which does not change with time. The skewing effect of the WT probably explains the significant genotype*time effect that we observed – which indicates that some genotypes follow a different progression to others. To establish this we also performed 2 way ANOVA on time vs genotype for MPS genotypes alone, which also confirmed that overall, the three MPS genotypes progress with time.

Our observations were confirmed by TEM analysis that showed an increased lysosomal burden and vacuoles, excess lipid storage, and dense cytoplasm in MPS cerebral cortex compared to WT. Furthermore, many more dystrophic axons containing autophagic organelles and storage material were present in the MPSIIIB brain compared to MPSI mice but none were detected in the MPSIIIA brain regions we analysed. These dystrophic axons were similar to those found in MPSIIIA mice by others [22] and in Alzheimer disease (AD), another neurodegenerative disease [31]. The dystrophic axons are comprised of vesicular organelles at various stages of autophagocytosis and may aid in the removal of damaged mitochondria so that initiation of apoptosis is avoided, resulting in a protective effect [31], [32].

Analysis of the disaccharide composition of HS from MPS brains indicated that the HS in all MPS types was abnormally highly sulphated compared to WT, with a trend for increased sulphation from MPSI to IIIA, with IIIB the highest. This effect was observed with a concomitant reduction in non- and mono-sulphated disaccharides in MPS brain compared to WT. This confirms our recently published work where significantly elevated levels of sulphated disaccharides were detected in MPSI brain at 3 months of age [17]. Elevated *N*-sulphated HS disaccharide levels have been found in MPSIIIA mouse brain compared to controls using tandem mass spectrometry [18], thus our results also support these observations. Furthermore, nuclear magnetic resonance and tandem mass spectrometry have shown elevated levels of HS and 2-*O*-sulphation in MPSI and IIIA patient urine and serum, with MPSIIIA exhibiting higher levels than MPSI [19], [20]. Interestingly, although the amounts of HS increased between MPSI and IIIA mice, significantly less HS appeared to be stored in MPSIIIB brains. This was surprising since MPSIIIB mice exhibited a significantly larger lysosomal compartment compared to MPSI and IIIA. The reason for this lower level of HS but larger lysosomal compartment is unclear. It could be due to excess storage of other materials such as cholesterol and other GM gangliosides in MPSIIIB brain compared to MPSI and IIIA, since GM2 ganglioside levels were similar in MPS brains. Alternatively, it could be specific to the AMAC assay, whereby the type of HS stored in MPSIIIB is not easily detected using this method or that this assay is subject to variability. These possibilities are currently under investigation.

The reasons why HS accumulation results in such severe brain dysfunction remains to be explained. The various combinations of *N*-, 6-*O*- and 2-*O*-sulphation modifications on HS are carried out by the activity of sulphotransferase enzymes and enable HS to be involved in many signalling processes. Highly sulphated HS is likely to have altered growth factor/morphogen binding capacity, increasing the binding of some factors while inhibiting the action of others [15], [33]. This may result in the persistence of any abnormal downstream signalling. Recently we have found that highly sulphated HS is responsible for changing one of the major pathways in haematopoietic stem cell homing in MPSIH (Watson *et al* unpublished data). Furthermore, we have shown that excess HS in MPSI augments HS sulphation by positively enhancing the sulphotransferase activity of *N*-deacetylase/*N*-sulphotransferase enzymes in the Golgi [17]. Thus it is not unreasonable to predict that excess HS in MPS diseases could play a significant role in perturbing HS mediated signalling pathways in the brain, thus perpetuating disease pathology.

It has been proposed that excess GAGs in the lysosome cause secondary storage by inhibiting ganglioside degrading enzymes [24], [34]. GM2 and GM3 ganglioside storage has been shown in mouse models of MPSI, IIIA, IIIB and VII [24]. We found that significant GM2 ganglioside storage had already occurred by 4 months of age with no significant increase at 9 months. The low level of residual enzyme activity (about 3–4% of WT) in the MPSIIIA model [22] does not appear to have any effect on the amount of gangliosides stored.

Since ganglioside storage is observed in the brains of patients with MPSI and MPSIIIA [35], [36] it has been proposed that secondary storage may play a role in disease progression [37]. In addition to the quantitative analysis of GM2 ganglioside immunoreactivity in the cerebral cortex, we also observed very intense GM2 ganglioside staining in other specific regions of the brain. These areas were i) the amygdala, which is thought to be responsible for memory and fear, ii) the lateral septal nucleus which is related to the control of locomoter activity and stress, iii) the thalamic and hypothalamic areas that direct and process signals from the motor cortex, and iv) the preoptic area which is involved in processing light and the circadian rhythm. McGlynn *et al* also detected GM2 ganglioside staining in similar areas including the cerebral cortex, amygdala, hippocampus and retrospenial cortex [24]. It is tempting to speculate that excessive GM2 gangliosidosis in these areas could contribute to behavioural changes in MPSIIIA [28] and IIIB [38] mice, including hyperactivity [28], [38], reduced sense of danger [28] and altered circadian rhythms [27], [39] that we and others observe. Many of the behavioural disturbances observed in mouse models of MPS correlate well with those in patients but it is also possible that other neuropathological factors are responsible for these observed behaviours.

A high level of neuroinflammation was reached by 4 months of age in all MPS mouse models, suggesting that this process starts early in life, in agreement with findings in MPSIIIB [25] and MPSIIIA brain [40]. Overall, astrocytosis was significantly higher in MPS compared to WT and increases with time in all MPS genotypes. Furthermore, astrocyte activation in MPSIIIA and IIIB was significantly higher compared to MPSI. When multiple comparisons were made between all genotypes at each timepoint, astrocyte activation in MPSI was significantly slower to progress compared to MPSIIIA and IIIB, but achieved similar levels by 9 months. However, microglial activation in MPSI mice was significantly lower than MPSIIIA and IIIB at both 4 and 9 months. To support this data, we found that a number of inflammatory cytokines associated with monocyte/macrophage (MIP-1α, MCP-1) and neutrophil (IL-1 α) recruitment to sites of inflammation were significantly elevated in MPS brains compared to WT. Furthermore, in MPSIIIB brain there was a significant increase in KC, also involved in neutrophil recruitment and G-CSF, which is involved in stimulation and proliferation of cells from the haematopoietic lineage. HS has been shown to play a major role in inflammation [41] and a number of studies have observed neuroinflammation in MPSI, IIIA and IIIB mouse models [21], [23], [25]. The elevated levels of highly sulphated HS that we have detected may be responsible for neuroinflammation. However, Ausseil *et al* have shown that in MPSIIIB mice deficient in Toll-like receptor 4 (TLR4), neurodegeneration can occur independently of microglial activation by HS, demonstrating that inflammation via this pathway is not responsible for the majority of pathology observed [21].

To further understand the cause of the neurodegeneration, we measured the cerebral cortex thickness and counted the number of neurons in specific areas of the cerebral cortex. A significant reduction in neuronal cell numbers was observed in MPSIIIA mice between 4–9 months, although no overall significant changes were observed between MPS and WT brains, thus casting doubt on the validity of this result. Vitry *et al* have also observed no significant changes in neuronal cell number or cerebral cortical thickness in MPSIIIB mouse brain compared to controls at 8 months of age [26]. It is possible that neuronal loss occurs at a later time point in mouse models of MPS compared to patients.

We detected a significant reduction in the level of VAMP2, but no change in the level of synaptophysin. These are synaptic vesicle associated membrane proteins involved in docking of vesicles at the pre-synaptic membrane, which forms part of the SNAP/SNARE complex, before neurotransmitter substance is released across the synapse allowing neurotransmission. VAMP2 staining was disorganised compared to WTs suggesting that the formation of synaptic vesicles or vesicle recycling may be disrupted by the increase in the lysosomal compartment and/or the defect in autophagy reported [42] in MPSIIIA. We have previously reported reduced and mislocalised VAMP2 staining in the suprachiasmatic nucleus of MPSIIIB mice [27]. VAMP2 knockout mice are not viable, but cells cultured from knockout embryos exhibit a defect in vesicle release [43]. Vitry *et al* detected a reduction in the level of synaptophysin in cerebral cortex layer I and II and overall, but no differences were observed in layers III, V and VI [26] which were more similar to the areas examined in this study (ie II/III–VI). Vitry *et al* also detected no differences in the level of VAMP in 8 month old MPSIIIB brains compared to WT. However, it was not stipulated whether they were detecting VAMP1 or 2 and there may be differences in areas of the cerebral cortex that were analysed [26].

Furthermore, we also detected a reduction in the level of Homer-1 in MPS mouse brain. Homer-1 is a scaffold protein enriched at post synaptic density of excitatory synapses where it binds glutamate receptors and allows targeting of downstream signaling through various pathways [44]. A reduction in Homer-1 may have downstream consequences on organisation and signaling at the post-synaptic density that may cause a defect in synaptic strength. Homer-1 knockout mice are viable but they display behavioural and neuronal defects [45], [46] and Homer signalling is found to be altered in several neurological disorders such as Schizophrenia, Alzheimer's disease, neuropathic pain, epilepsy and Fragile X syndrome [47]. It is evident that MPS patients exhibit progressive mental decline, behavioural difficulties, failure to reach developmental milestones and learning/memory deficits that may be due in part to degeneration of the synapse at the pre- and post-synaptic density.

What drives MPS disease progression still remains unclear. Many of the neuropathological measures progress from 4 to 9 months, albeit relatively slowly. However, this assumes that all MPS genotypes analysed here behave in a similar fashion. Thus, significant disease specific changes over time are only seen in MPSIIIB with lysosomal storage, MPSI with astrocyte activation and MPSIIIA with microgliosis and cortical neuronal loss whilst secondary pathology of GM2 appears to rapidly reach a threshold at 4 months in all diseases. To elucidate the progressive relationship between individual pathologies in each disease in more detail, earlier timepoints and greater mouse numbers would be required. It is clear that substantial pathology is already present in all genotypes by 4 months of age and despite progression in many factors the lack of overall change in cortical thickness and neuronal cell number, suggests that substantial neuronal damage has not yet occurred in mouse models of any of these diseases at either 4 or 9 months, despite clear behavioural abnormalities observed by us in MPSIIIA [28] and IIIB mice [38] by this age. Incidentally, behavioural abnormalities are much less established in both these models at 4 months of age, suggesting that disease progression is ongoing. Overall, in MPSI there appears to be less severe pathology in several parameters (the level of *2-O* HS sulphation, lysosomal compartment size, astrocyte activation and microgliosis) and disease progression to similar threshold values as MPSIIIA and IIIB from 4 to 9 months, suggesting that MPSI may have less severe overall neuropathology than MPSIIIA or IIIB, at least at these ages.

Neuroinflammation and neurodegeneration co-exist in a number of other brain degenerative lysosomal storage disorders [48], [49], [50] but it not known whether inflammation is a cause or consequence of neurodegeneration. It has been shown in a mouse model of multiple sclerosis, that prolonged exposure to TNF-α from microglia, for example, can cause degeneration of the synapse [51]. It is likely that in MPS diseases a combination of factors are responsible for neurodegeneration. The amount and length of exposure to different factors, as well as functional inhibition of cellular pathways are doubtless interlinked in these complex disorders. Aberrant biological activity of highly sulphated HS, prolonged inflammation, storage material affecting autophagy and other important cell functions such as synaptic vesicle trafficking, and potentially other unknown factors could all be combining to play a role in neurodegeneration.

In conclusion, we have evaluated and established baseline levels of a number of neuropathological markers of MPS disease that can be used as outcome measures for assessing new therapeutic strategies. The differences in severity of neuropathological events from MPSI to MPSIIIA and IIIB may have implications for the level of treatment required to correct the neuropathology observed.

## Methods

### Mouse maintenance and tissue collection

The mice used in this study were bred from heterozygotes, maintained on a C57BL/6J background and housed in a 12/12 hour light/dark cycle with food and water provided *ad libitum* in accordance with the Animal (Scientific Procedures) Act, 1986 (UK), project licence PPL 40/3056 and approved by the University of Manchester Ethical Review Process Committee. The MPSI and MPSIIIB mouse models are knockouts generated by targeted disruption of exon 6 in the *Idua* and *Naglu* genes respectively [52], [53]. The MPS IIIA mouse model is a spontaneously occurring mutant that retains around 3–4% of wild-type (WT) levels of SGSH activity [54]. WTs used in the study were a mixture of littermate controls from the 3 colonies and were a mix of males and females randomised between ages and genotypes. WT and MPS mice were sacrificed by cervical dislocation at 4 and 9 months of age. The brains were removed and either snap frozen and stored at −80°C for biochemical analysis or fixed in 4% paraformaldehyde in PBS for 24 hours at 4°C followed by cryopreservation in 30% sucrose, 2 mM MgCl_2_ in PBS for 48 hours at 4°C before storing at −80°C for histological analysis.

### Immunohistochemistry

Brain sections (30 µm) were cut using a freezing sledge microtome (Hyrax S30, Carl Zeiss, Hertfordshire, UK) and each section was stored in sequential wells of a 96 well plate for identification. Free floating immunohistochemistry was performed on sections taken from Bregma 0.26, −0.46 −1.18 and −1.94 mm according to the mouse brain atlas [55] and each subsequent antibody used the next adjacent set of comparative sections. Sections for each antibody were all stained on the same day using the same batch of solutions for each stain (n = 3 mice per group). Staining with antibodies against LAMP2 (developed by August, JT, Developmental Studies Hybridoma Bank, University of Iowa, USA), GFAP (1∶500; DakoCytomation, Ely, UK), VAMP2 (1∶500; Millipore, Watford, UK), synaptophysin (1∶200; Synaptic Systems, Gottingen, Germany) and Homer-1 (1∶2000; Synaptic Systems) were performed using the staining protocol as previously described [8], [27]. The microglia/macrophage staining was performed using peroxidase conjugated isolectin B4 from *Bandeiraea simplicifolia* (ILB4; Sigma, Poole, UK) as previously described [6], [27]. GM2 antibody (a gift from Dr Kostantin Dobrenis and Prof Walkley) staining was also performed as previously described [8], [24]. For LAMP2 and GM2 staining Nickel ions were included in the DAB substrate to produce a black stain for easier quantification. Sections were mounted onto positively charged slides (Fisher Scientific, Loughborough, UK) followed by clearing and mounting in DPX medium (Fisher Scientific). GFAP and ILB4 staining stained sections were counterstained with Mayer's haematoxylin before mounting.

For free floating immunofluorescent staining, sections were blocked with 10% goat serum, 1% Triton X-100, 1 mg/ml BSA in TBS for 1 hour at RT. Sections were incubated overnight at 4°C with LAMP2 and Alexa-594-labelled ILB4 or NeuN (Neuronal nuclei; Millipore, UK) diluted in blocking buffer. For ILB4 staining, 1 mM MgCl_2_ and CaCl_2_ were added to the buffers. Sections were washed 4 times with TBS and incubated with secondary antibodies, diluted 1∶1000 in blocking buffer, for 1 hour at RT (Alexa 594-goat anti-mouse IgG and Alexa 488-goat anti-rat IgG [1∶1000; Invitrogen, Paisley, UK]), followed by 300 nM DAPI (Invitrogen) for 15 minutes. Sections were washed with TBS (×4) and mounted onto positively charged slides with ProLong Gold Antifade mounting medium (Invitrogen). Sections were visualised using a Nikon C1 confocal on an upright 90 i microscope with a 60×/1.40 Plan Apo objective (Nikon Instruments Europe B.V., Kingston, UK).

### Image analysis

Four sections from each mouse brain (n = 3 mice per group), were imaged as follows. Two non-overlapping fields of view covering cerebral cortical layers II/III–VI per section were imaged using an Axioscop light microscope and Axiocam color CCD with Axiovision software (Carl Zeiss, Hertfordshire, UK) using the ×20 objective as shown in Figure 1A and 7A. The first image was taken by lining up the base of the field of view with the edge of the corpus callosum with the left edge in line with the apex of the cingulum so that the cortical layers were running in a horizontal direction. The second field of view was adjacent to the first field of view. These areas equate to the primary motor, somatosensory and parietal cortex. The observer was blinded to genotype and age. Images for each stain were all taken at the same exposure settings and in the same session. To quantify the antibody staining, the images were converted to 8 bits of grey resolution, saved in the TIFF format for analysis using Image J software (NIH, USA). For LAMP2 and GM2 staining, an unstained area was used to subtract background staining from each unmanipulated image. For each stain an average of the levels of optical density was calculated from 8 fields of view per mouse. The number of GFAP, ILB4 and Nissl positive cells were counted per image totalling 8 fields of view per mouse to produce an average number of cells per mouse. Nissl stained sections were also scanned using the Pannoramic SCAN, (Laser 2000 (UK), Ringstead, UK) and Laser2000 software was used to measure the cortical thickness. The first measurement was taken from the apex of the cingulum of the corpus callosum and the distance was measured to the outside of cerebral cortical layer II. The second measurement was taken from a point 1,000 µm laterally from the apex of the cingulum and the distance from the corpus callosum to the outside of cerebral cortical layer II was measured.

### Transmission Electron Microscopy

At 8–9 months of age, WT, MPSI, MPSIIIA and MPSIIIB mice (n = 3 mice per group) were transcardially perfused under anaesthesia (0.079 mg/ml fentanyl, 2.5 mg/ml fluanisone, 1.25 mg/ml midazolam) with Tyrode's buffer (138 mM NaCl, 1.8 mM CaCl_2_, 0.3 mM NaH_2_PO_4_, 5.6 mM glucose, 12 mM NaHCO_3_, 2.7 mM KCl, pH 7.4) followed by fixative (4% paraformaldehyde, 2% gluteraldehyde in 100 mM sodium cacodylate buffer pH 7.2–7.4). Brains were removed and placed in the same fixative for 1–2 hours at 4°C. A 1 mm coronal section was taken 3 mm from Bregma 2.8 and divided into 2 hemispheres using a mouse brain matrix. From the midline, a section of cortex (around 2 mm×3 mm) was taken from the corpus callosum up to the outside edge of the cerebral cortex was removed and processed as follows. Samples were washed for 5 minutes in 100 mM sodium cacodylate buffer and post-fixed in reduced osmium (1% OsO_4_+1.5% K4Fe(CN)6) in 100 mM sodium cacodylate buffer pH 7.2–7.4 for 1 hour at RT. Samples were incubated in 1% tannic acid in 0.1 M cacodylate buffer pH 7.2 for 1 h and in 1% uranyl acetate in water at RT for 1 hour followed by dehydration through an ethanol series (25%, 50%, 70%, 90% ethanol 10 minutes each) and absolute ethanol for 20 mins with 3 further changes (30 mins each). Samples were infiltrated with 50% TAAB LV resin/50% absolute alcohol for one hour, with 1 part absolute alcohol and 3 parts TAAB LVresin overnight then three fresh changes of 100% resin throughout the following day. Samples were embedded in fresh resin and polymerised at 60°C overnight. Sections (70 nm) were cut using Reichert Ultracut S ultramicrotome and visualised using a FEI Tecnai 12 Biotwin Transmission Electron Microscope at 80 kV acceleration voltage. Images were captured using a Gatan Orius SC1000 camera. Whole sections were assessed for gross morphological differences with an independent observer (n = 3 mice per group).

### Biochemical analysis of HS

Since a larger amount of starting material is required for this technique, one hemisphere of brain from 8–9 month old MPSI, MPSIIIA and MPSIIIB mice was used for HS biochemical analysis (n = 3 mice per group). Brain samples were disaggregated mechanically in PBS and treated essentially as described previously [17]. Briefly, tissues were pronase treated before GAGs were purified using a DEAE-sephacel column. Following desalting HS chains were digested into their component disaccharides using a combination of bacterial heparinases I, II and III enzymes. Resultant disaccharides were labelled with 2-aminoacridone (AMAC) and separated by RP-HPLC as described by Deakin and Lyon, applying the quoted disaccharide labelling efficiency factor during relative quantification [29]. Duplicate heparinase-digestions followed by RP-HPLC were performed per brain. Integration analysis of disaccharide peak-areas enabled relative quantification of HS amounts and disaccharide composition to be calculated. The percentage of total disaccharides containing either an *N*-acetylated or *N*-sulphated glucosamine, or containing 6-*O*-sulphation of GlcNAc or GlcNS or 2-*O*-sulphation of IduA or GlcA was also calculated from disaccharide compositions analyses, by summing the total number of disaccharides with that modification. An additional AMAC-labelled peak, that does not correspond with any known heparinase generated disaccharide was visible on the HPLC trace in MPSIIIA brain HS samples, at a unique location to the HS end structure previously identified in MPSI samples [17]. However due to its unknown structure it was excluded from disaccharide compositional calculations.

### Cytometric Bead Array

The levels of IL-1α, IL-1β, IL-3, IL-6, IL-9, IL-13, IFN-γ, MCP-1, MIP-1α, G-CSF, GM-CSF and KC (or CXCL1) were measured in whole brain extracts of 8–9 month old WT, MPSI, IIIA and IIIB mice (n = 5–6 per group) using BD Cytometric Bead Array (CBA) Flex Set kits (BD Biosciences, Oxford, UK). One hemisphere was homogenised in homogenisation buffer (250 µl; 50 mM Tris-HCl, 150 mM NaCl, 5 mM CaCl_2_, 0.02% NaN_3_, 1% Triton X-100, protease inhibitors, pH 7.4) using an electric homogeniser. Samples were centrifuged at 17,000 g at 4°C for 30 minutes and the supernatant used immediately in the CBA assay. A mix of standard beads for each flex set was reconstituted in Assay Diluent, to make a serial dilution for the standard curve (10–2500 pg/ml). The capture beads (0.2 ul per test) were mixed together in capture bead diluent (10 ul per test). The PE detection reagents (0.2 ul per test) were mixed together in detection reagent diluent (10 ul per test) and stored at 4°C in the dark until used. The mixed capture beads were mixed in an equal volume (10 ul) with standard or sample in FACS tubes (BD) and incubated at room temperature for 1 hour. This was followed by addition of 10 ul mixed detection reagent, and incubation at room temperature for 1 hour in the dark. Wash buffer was added and the samples centrifuged at 200 g for 5 minutes. The beads were resuspended in 200 ul wash buffer and vortexed before analysis on the flow cytometer (FACS Canto II, BD). The singlet bead population was identified on a FSC vs SSC plot, then the individual beads were separated using APC and APC-Cy7 and the level of each cytokine was measured on PE. 300 events were recorded per analyte, using the singlet population as the storage gate and stopping gate. The results were exported and analysed using FCAP Array software (BD). The protein concentration of the samples was measured using the BCA assay and the cytokine levels were standardised to protein level for each sample.

### Statistical analysis

All data was analysed using two way ANOVA with Tukey's multiple comparisons test to determine the differences between each group using JMP software (SAS Ltd, UK) except for the HS and CBA cytokine analysis which was analysed by one way ANOVA with Tukey's multiple comparisons test using SPSS software. Where significant genotype*time effects were seen in 2 way ANOVA, we established that WT was the genotype behaving differently to the MPS genotypes by performing a confirmatory 2 way ANOVA on time vs genotype for MPS genotypes alone. This allowed us to confirm that MPS genotypes all progress over time for some parameters.

## Supporting Information

Figure S1 **Lysosomal compartment size is significantly increased in MPS brain and localises to neurons and microglia.** LAMP2 (green) was detected in NeuN-positive neurons (red) and in ILB4-positive microglia (red) of layer II/III of WT, MPSI, IIIA and IIIB cerebral cortex. Nuclei are stained with DAPI (blue; Bar = 10 µm).
(TIF)

Figure S2 **Significant astrocytosis in MPS cerebral cortex at 4 and 9 months of age.** Representative sections of positively stained astrocytes (GFAP; brown) at 4 and 9 months of age (4 m and 9 m) that correspond to a whole field of view used for counting positive cells covering cortical layer IV (from section 2a, Figure 1A). Sections were counterstained with Mayer's haematoxylin to highlight the nuclei. Magnified sections are shown in Figure 5A. Bar = 100 µm.
(TIF)

Figure S3 **Significant microgliosis in MPS cerebral cortex at 4 and 9 months of age.** Representative sections of positively stained microglia (ILB4; brown) at 4 and 9 months of age (4 m and 9 m) that correspond to a whole field of view used for counting positive cells covering cortical layer IV (from section 2a, Figure 1A). Sections were counterstained with Mayer's haematoxylin to highlight the nuclei. Magnified sections are shown in Figure 5C. Bar = 100 µm.
(TIF)
