# Supplementary figures and images for: Neuropathology in Mouse Models of Mucopolysaccharidosis Type I, IIIA and IIIB
Source: PLoS One. 2012 Apr 27;7(4):e35787. doi: 10.1371/journal.pone.0035787 (PMC3338781; doi:10.1371/journal.pone.0035787)

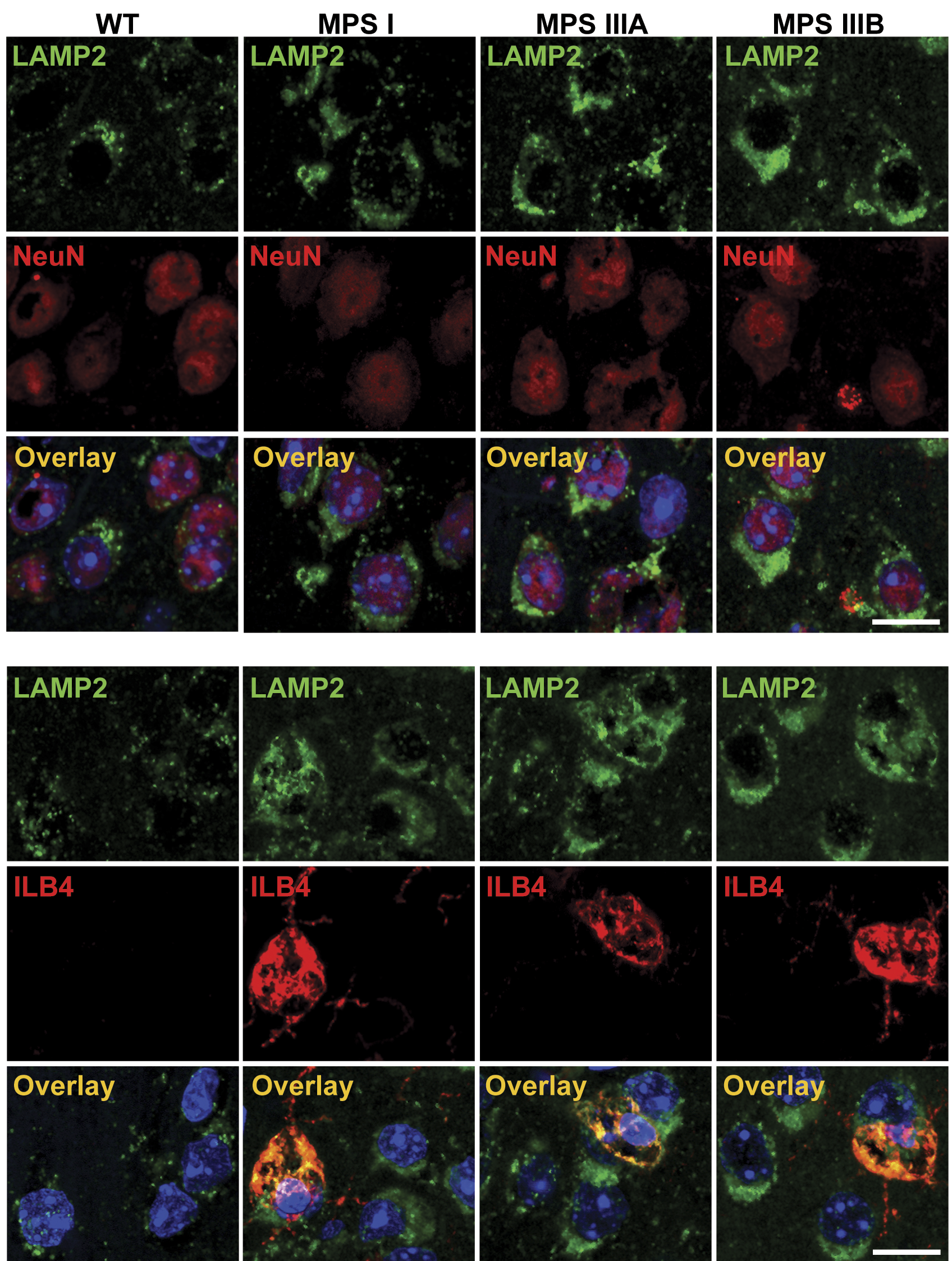

Supplement: Figure S1 — Lysosomal compartment size is significantly increased in MPS brain and localises to neurons and microglia. LAMP2 (green) was detected in NeuN-positive neurons (red) and in ILB4-positive microglia (red) of layer II/III of WT, MPSI, IIIA and IIIB cerebral cortex. Nuclei are stained with DAPI (blue; Bar = 10 µm). (TIF) [file pone.0035787.s001.tif]

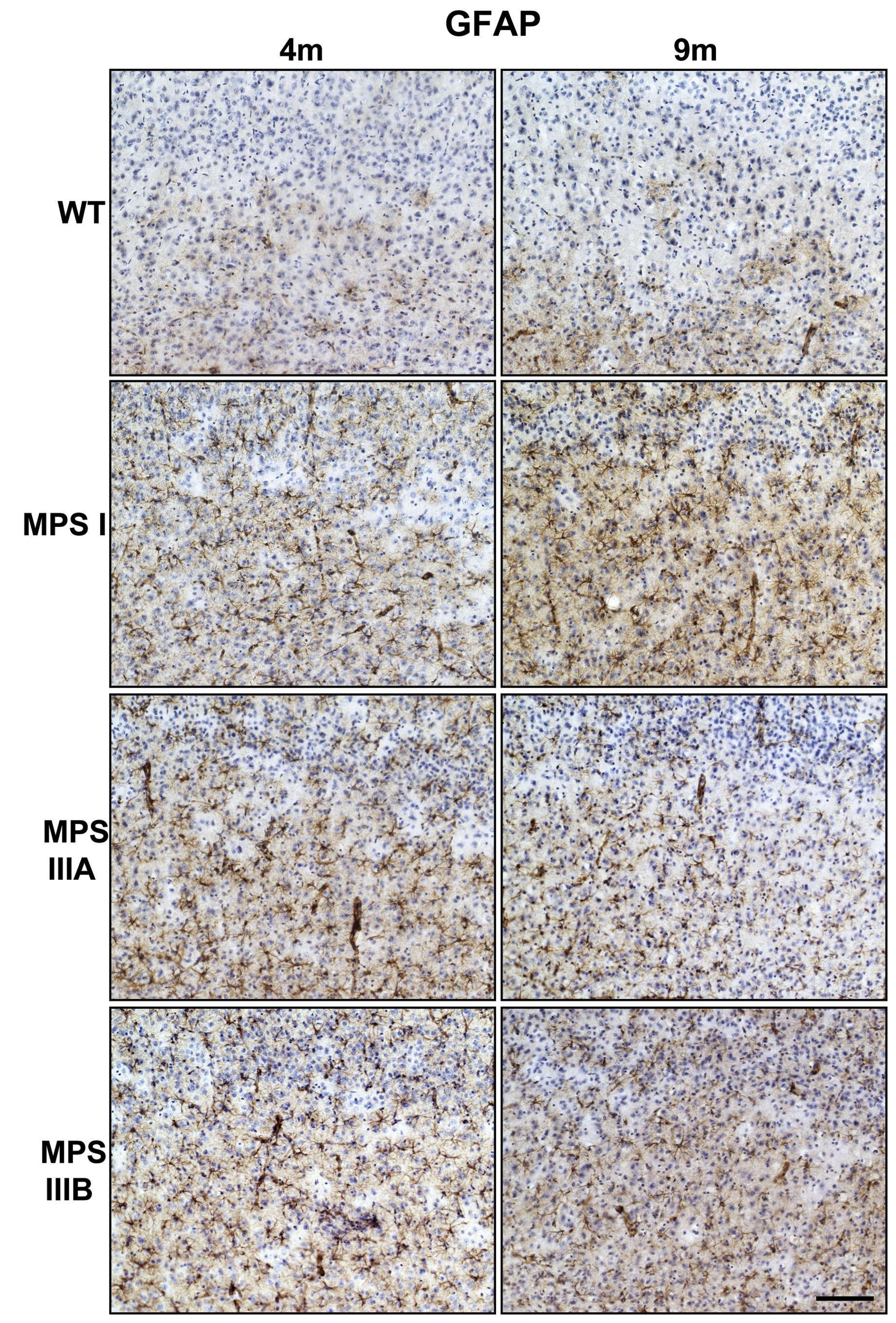

Supplement: Figure S2 — Significant astrocytosis in MPS cerebral cortex at 4 and 9 months of age. Representative sections of positively stained astrocytes (GFAP; brown) at 4 and 9 months of age (4 m and 9 m) that correspond to a whole field of view used for counting positive cells covering cortical layer IV (from section 2a, Figure 1A). Sections were counterstained with Mayer's haematoxylin to highlight the nuclei. Magnified sections are shown in Figure 5A. Bar = 100 µm. (TIF) [file pone.0035787.s002.tif]

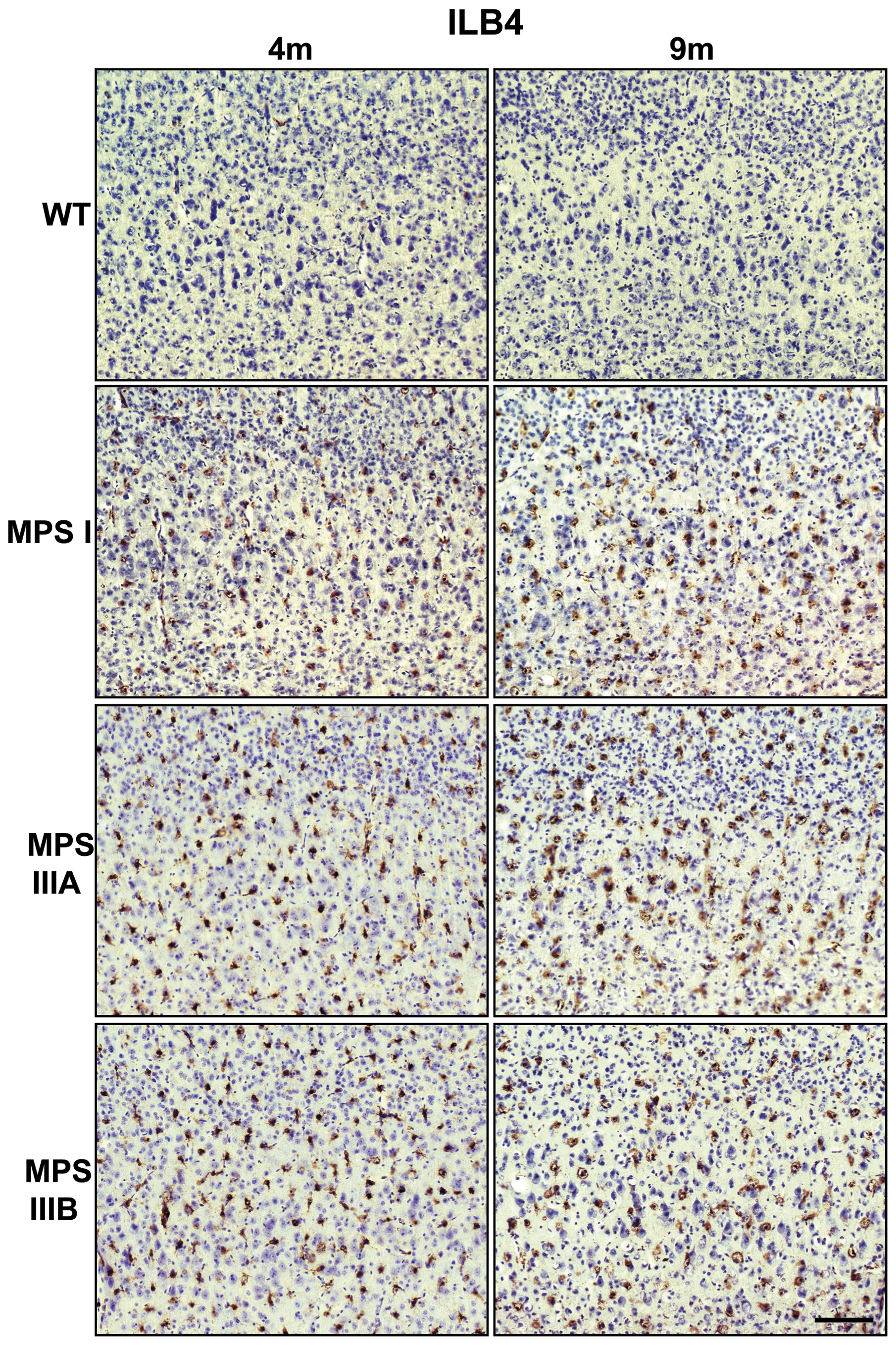

Supplement: Figure S3 — Significant microgliosis in MPS cerebral cortex at 4 and 9 months of age. Representative sections of positively stained microglia (ILB4; brown) at 4 and 9 months of age (4 m and 9 m) that correspond to a whole field of view used for counting positive cells covering cortical layer IV (from section 2a, Figure 1A). Sections were counterstained with Mayer's haematoxylin to highlight the nuclei. Magnified sections are shown in Figure 5C. Bar = 100 µm. (TIF) [file pone.0035787.s003.tif]
